# Restricted mobility of specific functional groups reduces anti-cancer drug activity in healthy cells

**DOI:** 10.1038/srep22478

**Published:** 2016-03-02

**Authors:** Murillo L. Martins, Rosanna Ignazzi, Juergen Eckert, Benjamin Watts, Ramon Kaneno, Willian F. Zambuzzi, Luke Daemen, Margarida J. Saeki, Heloisa N. Bordallo

**Affiliations:** 1 Niels Bohr Institute, University of Copenhagen, DK-2100 Copenhagen, Denmark; 2 Instituto de Biociências - Universidade Estadual Paulista – CP 510, 18618-970 Botucatu–SP, Brazil; 3 Department of Chemistry, University of South Florida, 4202 E. Fowler Ave., Tampa, Florida 33620, United States; 4 Los Alamos National Laboratory, Los Alamos, New Mexico 87545, United States; 5 Swiss Light Source, Paul Scherrer Institute, CH-5232 Villigen, Switzerland; 6 European Spallation Source ESS AB, PO Box 176, SE-221 00 Lund, Sweden

## Abstract

The most common cancer treatments currently available are radio- and chemo-therapy. These therapies have, however, drawbacks, such as, the reduction in quality of life and the low efficiency of radiotherapy in cases of multiple metastases. To lessen these effects, we have encapsulated an anti-cancer drug into a biocompatible matrix. *In-vitro* assays indicate that this bio-nanocomposite is able to interact and cause morphological changes in cancer cells. Meanwhile, no alterations were observed in monocytes and fibroblasts, indicating that this system might carry the drug in living organisms with reduced clearance rate and toxicity. X-rays and neutrons were used to investigate the carrier structure, as well as to assess the drug mobility within the bio-nanocomposite. From these unique data we show that partial mobility restriction of active groups of the drug molecule suggests why this carrier design is potentially safer to healthy cells.

Cancer is one of the main worldwide public health concerns. In Europe, the incidence of this disease has increased from 3.2 million new cases in 2008 to 3.45 million in 2012, with a mortality rate around 50%^1^,^2^. Paclitaxel (PTX) is one of the most effective drugs currently available for the treatment of breast, lung and ovarian cancers^3^,^4^,^5^,^6^. Its function is based on a unique mechanism involving the stabilization of cell microtubules, which explains its therapeutic success^7^. However, considerable limitations still exist regarding this drug, mainly due to its low water solubility (~0.4 μg/mL) and, of course, its toxicity to healthy cells. To increase its solubility, a drug is often formulated in organic solvents, such as dehydrated ethanol and polyoxyethylated castor oil. Unfortunately this approach causes many side effects, such as hypersensitivity reactions and hyperlipidaemia^8^.

Consequently the development or modification of systems for accommodating and delivering anti-cancer drugs is of utmost importance^9^. A promising alternative is the use of soluble polymeric nano-carriers for controlling the pharmacokinetic and bio-distribution of the drug^10^. The biopolymer chitosan, in particular, has attracted great interest in biomedical applications because of its biocompatibility and biodegradability^11^. This path has also been used as an encapsulation matrix for PTX with promising results^12^,^13^. Further improvements can be made by modifying the surface features of the drug delivery system with low toxicity compounds, which may also make possible to increase the adhesion of the carrier to cancer cells^14^. To this end, the use of hydroxyapatite (Ca_10_(PO_4_)_6_(OH)_2_, hereafter HAP), the main inorganic constituent of human bones and teeth, is an excellent candidate. At the nano-scale, HAP presents special biocompatibility as well as non-immunogenicity, non-inflammatory behaviour, high osteoconductivity and good adhesion to different types of cancer cells^15^,^16^. Of even more interest, HAP nanoparticles (nHAP) show inhibitory effect on cancer cells proliferation with lower effects on the healthy ones^16^,^17^,^18^,^19^. Consequently, the combination of the properties of nHAP with biopolymers in a nano-composite, may lead to drug delivery systems with inherent effects on cancer cells. However, to take full advantage of the nHAP properties, these nanoparticles must be in the out layer of the composite^20^. Additionally to the benefits derived from combining a biopolymer with nHAP, inclusion of a drug into nano-carriers with magnetic properties, for instance Mn-Zn ferrite nanoparticles, offers remarkable new possibilities. For example, guiding the drug carrier along the body using external magnetic field as well as monitoring its position by gradiometers or magnetic resonance imaging^21^,^22^,^23^,^24^. Finally, magnetic hyperthermia treatments, which represent a promising technique used in combination with radio and chemotherapy, also become practicable^25^,^26^,^27^.

Following the ideas described above, we have encapsulated PTX into a bio-nanocomposite (hereafter bio-NCP and bio-NCP + PTX) formed by Mn-Zn ferrite nanoparticles coated with chitosan, which surface was modified with nHAP^28^.

Morphological *in-vitro* tests, performed using scanning electron microscopy (SEM) and energy dispersive X-ray spectroscopy (EDS), based on a methodology developed by our group, allowed for a preliminary insight on the interaction between cells and nanoparticles without the need of fluorescent or radioactive markers in the nanoparticles. The results suggested that normal monocytes and two distinct types of tumour cells interact differently with the bio-NCP. While no toxicity was observed on the healthy cells, morphological changes were detected mainly in colon cancer cells.

The rather challenging characterization of the formulated bio-NCP + PTX and insight into the dynamics of the encapsulated and released drug were achieved by using advanced microscopy and spectroscopy techniques, respectively. These include near edge X-ray absorption fine structure (NEXAFS) spectroscopy, scanning transmission X-ray microscopy (STXM) and inelastic neutron scattering (INS).

NEXAFS made possible to characterize different organic groups on account of the interaction of X-rays with the K-shell of the carbon atoms, without the magnetic nanoparticles affecting the result^29^. By combining NEXAFS with STXM, the chemical compositional map along with the visual analysis of the PTX distribution of the bio-NCP was obtained. These results indicate that PTX is distributed within the polymeric part of the carrier.

Finally, the comparison of the dynamics of the encapsulated drug to that of the pure form–a key step in understanding and controlling the polymer/drug interactions and one of the major questions in the further development of this technology towards clinical trials–was obtained by combining INS with Density Functional Theory (DFT) calculations. These results provided for the assignment of the vibrational modes, including those observed within the drug carrier^30^,^31^,^32^. Using this approach, we show that although the phenyl and acetyl vibrational modes are constrained by the encapsulation, they seem to be recovered after the drug release. This is important because the PTX activity is known to be related to the mobility of these groups^7^.

To conclude, the ensemble of our results indicates that the proposed bio-NCP can open new opportunities for the emerging field of drug delivery.

## Results

### Monocytes reaction inhibited by the hydroxyapatite modification

The potential of the bio-NCP as a PTX carrier is highlighted by the *in-vitro* tests with monocytes, which tend to promote phagocytosis in foreign particles or molecules in the human body, blocking them to reach target tissues.

**Figure 1:**
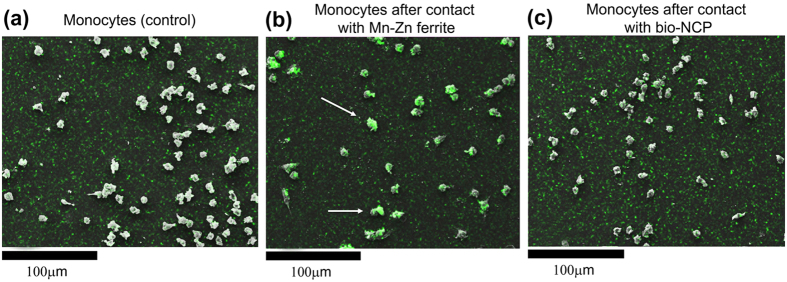
Representative SEM images and EDS analyses of normal monocytes of a healthy donor (control group) (**a**) and the cells after being in contact for 2 h either with Mn-Zn ferrite nanoparticles (**b**) or with the bio-NCP (**c**). High Fe concentrations, marked green, suggest uptake from the Mn-Zn ferrite nanoparticles.

Morphological changes of monocytes from a healthy donor (control group) (Fig. 1(a)) were visually evaluated in response to their contact for 2 h with the pure ferrite nanoparticles (Fig. 1(b)) and with the bio-NCP (Fig. 1(c)) by means of SEM. In both cases, considerable morphological changes were not observed. Subsequently, the cells were analysed by EDS in order to determine regions with high Fe concentration, the main component of the Mn-Zn ferrite nanoparticles that composes the core of the bio-NCP. Such observation provides insight on the interactions between the cells and the nanoparticles. This is indeed the case in the assay with Mn-Zn ferrite, where cells with high Fe concentration, marked green and highlighted by the arrows in the representative SEM micrograph, were observed as shown in Fig. 1(b). This scenario changes by modifying the nanoparticles with the bio-NCP, where the cells present smaller Fe concentration after the assay as shown in Fig. 1(c). Thus suggesting that the bio-polymeric coating modified with nHAP inhibits the reaction from the defence cells, which is known to compromise the function of the carrier^33^.

### Morphological changes observed on cancer cell lines and cytotoxicity tests in fibroblasts

**Figure 2:**
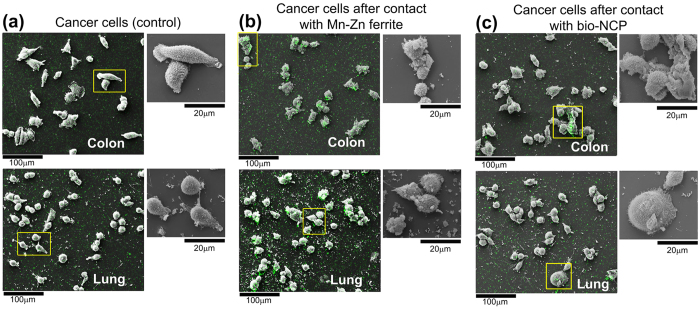
Representative SEM images and EDS analyses of in-vitro analyses of colon (HCT116) and lung (3LL) cancer cells (control) (**a**) and the cells after 2 h contact with Mn-Zn ferrite (**b**) and bio-NCP (**c**). The green spots depict regions with high Fe concentration as determined by EDS and the insets present zoomed images of the selected regions. The colon cancer cells present the most evident morphological changes after contact with both Mn-Zn ferrite and the bio-NCP.

Figure 2(a) presents representative SEM images of control groups of colon (top) and lung (bottom) cancer cells, while Fig. 2(bc) present the cells after 2 h contact with Mn-Zn ferrite and bio-NCP nanoparticles, respectively. The cells were also subjected to EDS analyses as depicted by the green spots, which are more evident for the cells tested with pure Mn-Zn ferrite nanoparticles, Fig. 2(b).

**Figure 3:**
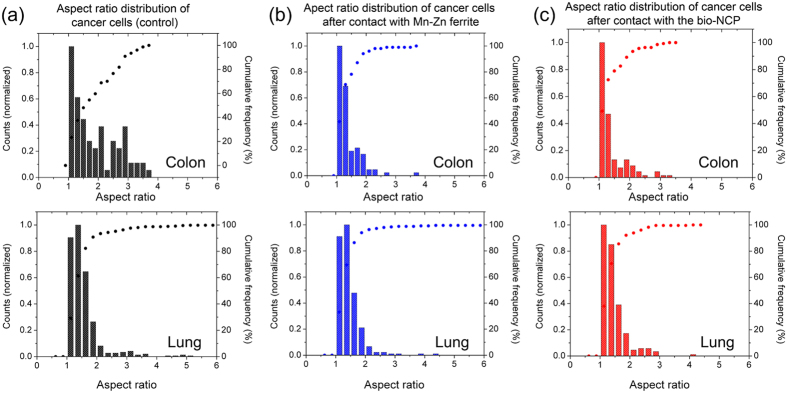
Aspect ratio distributions represented by bars for (**a**) colon (HCT116) and lung (3LL) cancer control cells and for the cells after 2 h contact with (**b**) Mn-Zn ferrite and (**c**) bio-NCP. The morphological changes, more evident in the colon cancer cells after contact with both Mn-Zn ferrite and the bio-NCP, are reflected by the sharper distributions. In each figure the symbols represent the cumulative frequency of the aspect-ratios.

Additionally, in each figure the insets show zoomed images of selected areas to highlight cellular morphological changes. These changes were evaluated by comparing the distribution of the cells aspect ratio, which refers to the ratio between the major and minor axis of an ellipse describing the shape of each cell, before and after their contact with the Mn-Zn ferrite and the bio-NCP nanoparticles. Under these lines, aspect ratio distributions closer to 1 indicate cells with predominantly spherical shapes. These results are shown in Fig. 3. From these analyses one obtains a mean aspect ratios of 1.9 and 1.5 for the colon and lung control groups, respectively. Interestingly, after contact between the colon cancer cells and both Mn-Zn ferrite and the bio-NCP the distribution of the cells aspect ratio showed a decrease of about 26%, reaching a mean value of 1.4. No detectable changes are detected for the lung cells.

**Figure 4:**
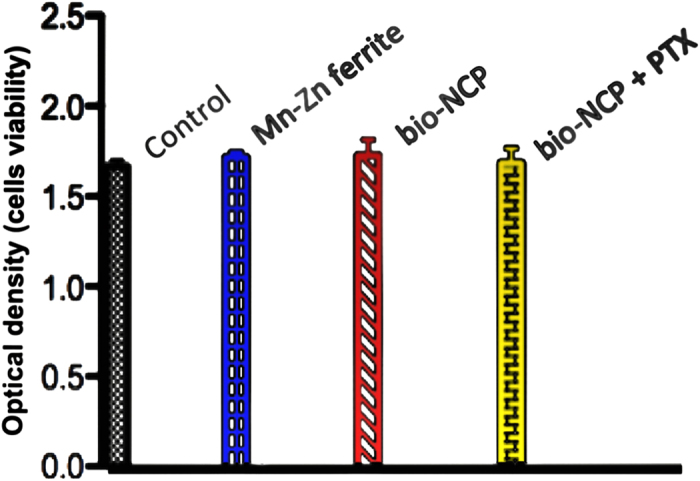
Viability test on fibroblast after 24 h in contact with the Mn-Zn ferrite nanoparticles, the bio-NCP and the complex bio-NCP + PTX showing no significant toxicity. Note that the control sample is the fibroblast without contact with any material.

Finally, a preliminary cytotoxicity test of the materials, including the bio-NCP + PTX, to healthy cells was provided by *in-vitro* assays with fibroblasts, adopted here as a model for healthy cells, following the recommendation given by ISO 10 993-5. As presented in Fig. 4, the fibroblasts viability after the assays exhibits no significant difference in comparison with the results obtained for the control group, indicating no significant toxicity.

### Chemical compositional map of the drug carrier and confirmation of successful encapsulation of the drug

**Figure 5:**
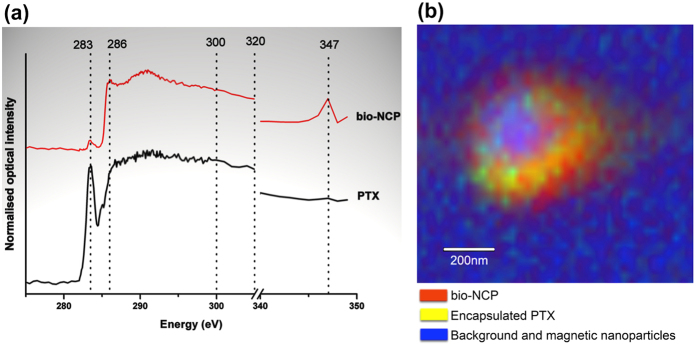
NEXAFS spectra for PTX and the bio-NCP (**a**). The PTX spectrum shows a characteristic peak at 283 eV, while the bio-NCP spectrum shows characteristic transitions at 286 eV, 291 eV and 347 eV. In (**b**), the map of the chemical composition of the bio-NCP + PTX obtained by STXM after performing singular value decomposition on images collected using X-rays with the following energies: 275 eV, 283 eV, 286 eV, 300 eV, 320 eV and 347 eV. The PTX is represented in yellow, the bio-NCP in red and the blue colour corresponds to background materials, including the magnetic nanoparticles. The green spots denote regions with low PTX concentration. The compositional map shows the distribution of the PTX in the polymeric nHAP shell.

NEXAFS spectra for PTX and bio-NCP, presented in Fig. 5(a), are broadened by the convolution of excitations on account of the considerable flexibility of the carbon bonds^34^. Nonetheless, the PTX fingerprint at 283.3 eV is clearly detected and can be related to a C-C π*-bond, which is characteristic of aromatic rings. A peak at 286 eV in the spectrum of bio-NCP may be related to a C1s → σ* transition on the C-OH bonds, while the broad band at 291 eV is assigned to the excitation of C, H and N bonds from the cross-linked chitosan^34^. The noteworthy peak in the bio-NCP spectrum at 347 eV is related to the Ca L_3,2_-edge and indicates the apatite modification on the chitosan surface^35^.

Figure 5(b) presents the chemical composition map derived from the STXM data. In this map the PTX is represented by yellow and the bio-NCP by red. The blue colour represents the background materials, including the magnetic nanoparticles. The green spots, a mixture of yellow and blue, denote regions with low PTX concentration. The resulting image shows that the drug is distributed along the chitosan/nHAP shell with the Mn-Zn ferrite magnetic nanoparticles forming the core of the bio-nanocomposite. This distribution confirms the successful encapsulation of the drug.

### Initial studies of the releasing process

To verify the recovery of PTX after exposure of the bio-NCP + PTX in aqueous media for longer times, the latter was dispersed in water at 37 °C (human body temperature) for 7 days, dried at room temperature under vacuum and investigated by FTIR. All spectral features specific to the dispersed sample, bio-NCP + PTX as prepared and for the pure PTX are compared to the results of the DFT calculations for the gas phase molecule and discussed in detail in the supplementary information. The most striking result was the observation of the modes located at 1535 and 1550 cm^−1^, assigned to benzene rings. These modes are recovered after the drug release and related to the anti-tumour activity of PTX^7^.

### Insights on the interactions of the drug with its carrier

The next key point for elucidating the releasing process was the analysis of the interactions of the drug with its carrier. This step was accomplished by combining INS experiments with DFT calculations on the free molecule. Details on these calculations, assisted with the full identification of the vibrational features in the spectra by animations for the pure and encapsulated drug are given in the supplementary information. It is worth noticing that the calculations are performed in the harmonic approximation, while the vibrational modes of our materials, particularly the low frequencies ones, are likely to be anharmonic modes. The schematic structure of the PTX molecule adapted from^7^ is also depicted in the supplementary information.

**Figure 6:**
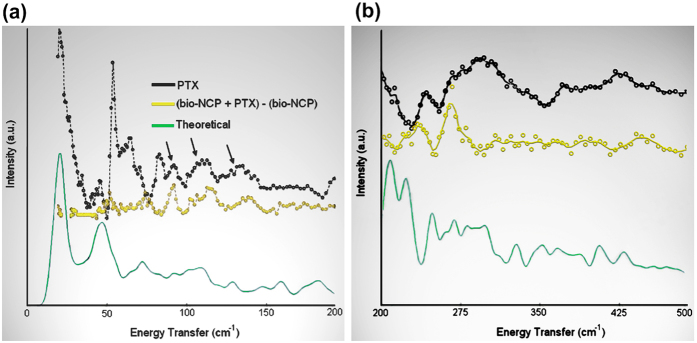
INS data collected at FDS between (**a**) 20 and 200 cm^−1^ and (**b**) between 200 and 500 cm^−1^. In (**a**,**b**) the black curve shows the PTX data, while the contribution from the encapsulated drug, shown in yellow, is depicted by the difference spectrum between the (bio-NCP + PTX) and (bio-NCP) spectra. The bio-NCP data is not shown. INS spectra obtained by DFT calculations for the free molecule are shown in green. In the difference spectrum, the modes assigned to acetyl and phenyl groups, observed below 80 cm^−1^, are not detected, while vibrations from the terpene ring, indicated by arrows, remain visible in the encapsulated drug. This indicates that the mobility of the biologically active groups of PTX is highly constrained.

In Fig. 6(a), the sharp maximum around 56 cm^−1^ is related to the acoustic mode for H_2_O^36^. Here we should recall that the PTX used in this work is a mixture of hydrated and dehydrated forms^37^. The modes above 60 cm^−1^ are assigned to acetyl and phenyl groups in the PTX molecule are not detected in the (bio-NCP + PTX-bio-NCP) spectrum, which corresponds to that of the encapsulated PTX molecule. This indicates that either the molecule is severely constrained or has adapted a new conformation. However, it is also clear, as showed by arrows, that some higher frequencies vibrations assigned to the PTX molecule do remain in the (bio-NCP + PTX-bio-NCP) spectrum. These vibrations originate mainly from carbons in the terpene ring itself as well as from lattice motions, implying that the rigid structure of PTX is maintained even after encapsulation. As shown in Fig. 6(b), remaining methyl vibrations are also detected between 200 cm^−1^ and 270 cm^−1^, while the modes above 300 cm^−1^ appear weak on account of poor statistics from the subtraction procedure. Therefore we are led to conclude that the restraining of the acetyl and phenyl groups is most likely due to the folding of the molecule.

## Discussion

Our first *in-vitro* assays show that no morphological changes were observed in monocytes after their contact with the pure Mn-Zn ferrite nanoparticles or with the bio-NCP. After contact with the Mn-Zn ferrite, however, several monocytes presented high Fe concentration. The latter observation indicates interaction and/or uptake of the magnetic nanoparticles by the monocytes. Meanwhile, very low Fe concentration was detected in the tests performed with the bio-NCP, which could be, in principle a consequence of the lower Fe concentration in comparison to the pure ferrite. However, given the set up of this experiment, described in the Methods Section, it is plausible to consider that the bio-NCP inhibits the interaction/uptake of the monocytes. Further *in-vitro* assays performed with colon and lung cancer cells indicate that both the Mn-Zn ferrite nanoparticles and the bio-NCP interact and cause morphological changes in cancer cells, especially those from the colon. This assumption is drawn based on the variation of the aspect ratio distribution, which was initially characteristic of elongated cells, and became closer to that describing spherical ones after the interaction. Even in an early stage, this is an encouraging result, since the invasiveness of colon cancer cell lines has been associated with its elongated morphology^38^. Additionally, as the response observed for both Mn-Zn ferrite nanoparticles and the bio-NCP are very similar, it is reasonable to hypothesize that the bio-NCP effect has its origin on the magnetic core. Indeed, effects of Fe-based nanoparticles on cancer cells, including those from the colon, have been attributed to controlled Fe-catalyzed reactive oxygen species (ROS) that potentially trigger autophagy and associated cell death^39^,^40^. Consequently, this observation seems to suggest that Fe ions are released through the bio-NCP’s polymer/apatite shell during this particular experiment. On the other hand, the lack of toxicity to fibroblasts can be related to the reduction of Fe toxicity to healthy cells at low Fe concentrations^41^,^42^. These are motivating results for the application of the bio-NCP + PTX in cancer treatment, since it seems to be non-toxic to healthy cells. However, these results also suggest that the drug is either in a very low concentration into the nanocomposite or it is not readily released under the experimental conditions of the chosen protocol. To answer this question it is necessary to investigate the PTX concentration into the bio-NCP. A task not at all straightforward due to the complexity of the bio-NCP + PTX, which makes the application of the most common techniques, such as HPLC (high performance liquid chromatography), challenging and creates the need to use more sophisticated solid-state approaches. Therefore, by combining NEXAFS and STXM a map of the chemical composition was obtained for the bio-NCP + PTX. This map shows that a significant amount of the drug is indeed distributed within the chitosan and the nHAP shell, suggesting that the slow PTX release is most likely due to its confinement in the matrix.

The latter observation pointed us to the need of understanding how the confinement influences the PTX dynamics. This question was answered by combining INS and DFT. The simplicity of the neutron−nucleus interaction and the exceptionally high incoherent scattering cross-section of the hydrogen atom compared to that of any other element were key to such understanding^43^. From such analysis we concluded that the mobility of the biologically active groups of PTX, i.e. acetyl and phenyl groups, are highly constrained in the bio-NCP + PTX. Therefore, one can hypothesize that the carrier limits the PTX’s activity not only as a physical barrier between the drug and the action sites, but also by restricting motions on specific parts of the molecule. However, after release, these vibrational motions were partially recovered, as observed by means of FTIR, indicating that the anti-cancer agent might regain its active form.

From the viability and INS results it is now clear that before envisaging future applications of the bio-NCP + PTX in *in-vivo* trials, the drug release rate needs to be better controlled. One obvious approach to circumvent the problem would be the modification of the bio-NCP structure, i.e the cross-linking and mimetization degree over the chitosan, or even the choice of another polymer with a different surface charge distribution, such as poly-ethylene glycol^44^. Nonetheless before progressing in this direction, few questions need to be further considered, such as the evaluation of the release behavior of PTX from the bio-NCP in the surroundings of cancer cells. The inherent slightly lower pH environment compared to that of healthy cells could be enough to degrade or relax the chitosan network^45^,^46^. In addition, the use of radiofrequency as another possibility ought to be investigated. In this case, by heating the magnetic nanoparticles^27^ the polymeric network can be relaxed and in turn the drug release could be facilitated.

In conclusion, we report on encouraging results on the application of a new bio-NCP as a PTX carrier. We also propose new methodologies for the study of encapsulated drugs, based on state-of-the-art scattering techniques combined with theoretical calculations. The next steps in this work will focus on further understanding the encapsulation effects on the dynamics of the released PTX and its correlation with its biological activity as well as in the optimization of the drug release mechanism.

## Methods

### Sample preparation

The detailed synthesis process for obtaining the bio-NCP is described elsewhere^28^. Briefly, from neutron diffraction results^28^, we determined that 0.41 mg of Fe per mg of nanoparticles were precipitated from a solution of salts, and subsequently encapsulated in chitosan by the double emulsion method. The chitosan was then cross-linked by reaction with glutaraldehyde and a final mimetization process was performed to modify the surface with apatite forming the bio-NCP nanocomposite. Depending on the mimetization efficiency, which is not easily determined as it is connected to the amount of apatite effectively present in the polymer surface, in the final bio-NCP the amount of Fe per mg can vary between 0.20 mg (100% efficiency) to 0.27 mg (0% efficiency). The bio-NCP + PTX was produced by a similar method that included adding PTX to the solution before crosslinking of the chitosan. Therefore we can assume that the amount of Fe content is the bio-NCP + PTX is similar. Further details are given in the supplementary information.

### *In-vitro* tests using human monocytes, HCT116 (colon cancer) and 3LL (lung cancer) cultures

Human monocytes were isolated from peripheral blood of healthy donors in accordance to the Declaration of Helsinki. All healthy volunteers provided informed consent. The study was approved by the Health Research Ethics Committee, Plataforma Brasil (http://aplicacao.saude.gov.br/plataformabrasil/login.jsf), with the decision number 1358038 and the CAAE number 50995715.9.0000.5411. Stable cancer cell lines, provided by Dr. Kaneno’s laboratory, were used. Thus no approval from the local ethical committee was required. The detailed processes involved on the preparation of cell cultures are described in the supplementary information. After preparation, the cells were set to 2 × 10^5^ cells/mL and dispensed on rounded glass slides (∅13 mm), previously coated with poly-L-lysine. To promote the cell attachment on the slides, the cultures were kept for 2 h at 37 °C under 5% CO_2_.

### *In-vitro* response of monocytes, *HCT116 and 3LL cells* to pure ferrite and bio-NCP nanoparticles

The glass slides were washed three times with warm complete culture medium (described in supplementary information) (1 mL each time) and allowed to interact with 50 μg of either pure ferrite or bio-NCP nanoparticles. The cell cultures were kept for 2 h at 37 °C under 5% CO_2_ to allow direct contact between the cell surfaces and the nanoparticles. The slides were then washed with fresh phosphate buffered salt solution (PBS) at room temperature, the cell monolayers fixed with 2.5% glutaraldehyde and routinely processed to microscopy analysis as follows.

### Morphological analysis by scanning electron microscopy

After fixation of the monolayers on the slides, the cells were dehydrated with ethanol solutions of 7.5, 15, 30, 50, 70, 90 and 100%, twice within 10 min for each alcohol concentration, and submitted to supercritical drying (over the critical point) in a CO_2_ atmosphere for further metallization. The samples were subsequently analysed with scanning electron microscopy (SEM) (FEI, Quanta 200 equipped with an Oxford, Inca, 250P20 EDS) and 7 images (1000 × magnification) were randomly collected for each slide. The distributions of the cells aspect ratio were evaluated with the software ImagePro 4.1.0.0. No study was conducted for determining the number of cells; therefore the cell counts were normalized to the maximum value. EDS was used to map the Fe concentration over the cells. The nanoparticles amount, as well as the instrument parameters were set to provide the same signal/noise ratio for a same mass of either Mn-Zn ferrite or bio-NCP.

### Cytotoxicity assays with Balb/c 3T3 fibroblasts

The toxicity of the pure ferrite, bio-NCP and bio-NCP  +  PTX was evaluated in respect to Balb/c 3T3 fibroblasts (clone A31–American Type Culture Collection) as follows. Firstly, the cells were processed as described in the supplementary information, dispensed in 96-well plates, each one containing 5 × 10^4^ cells, and kept in culture for 24 h. Subsequently, 50 μg of each sample, i.e. pure ferrite, bio-NCP and bio-NCP  +  PTX, was suspended in the same culture media used to cultivate the cells and added to the plates allowing for their interaction with the cells. After 24 h, the supernatant, i.e. the excess of liquid and magnetic nanoparticles, was removed, the cells washed with PBS-A and subjected to incubation with 3-[4,5-dimethylthiazol-2-yl]-2,5-diphenyltetrazolium bromide (MTT) with 0.5 mg of MTT/mL of DMEM for 4 hours. This resulted in the formation of the formazan dye in the living cells, which was solubilized in DMSO and quantified using a microplate photometer by reading at 570 nm. The obtained absorbance values reflect the viability of the cells. The experiments were performed in duplicates with a n = 6 each.

### Near edge X-ray absorption fine structure (NEXAFS) spectroscopy and Scanning Transmission X-ray Microscopy (STXM)

PTX and bio-NCP samples were dispersed in ethanol for deposition onto silicon nitride membranes (Silson, England) and placed in a low-vacuum environment for X-ray analysis using the carbon K-edge photon energy range (250 to 350 eV) at the PolLux beam-line (Swiss Light Source at the Paul Scherrer Institute, Switzerland)^47^,^48^,^49^. For the STXM experiments, a monochromatic X-ray beam is focused on the sample and the measured transmitted intensity is used to build a pixel-by-pixel image. When tuning the X-ray photon energy to resonance features present in the NEXAFS spectrum, the STXM images will display strong features that are related to the natural contrast based on the molecular bonding of the constituent materials. Thus by combining a set of images obtained at selected photon energies with the NEXAFS spectra of the component materials, the sample chemical composition map is calculated using singular value decomposition^50^. Therefore, after the NEXAFS spectra of the PTX and of the bio-NCP were collected, STXM images for the bio-NCP + PTX were obtained at X-ray energies of: 275 eV, 283 eV, 286 eV, 300 eV, 320 eV and 347 eV. The images and spectra were analysed using the aXis2000 software package^51^.

### Inelastic neutron scattering (INS) and Density Functional Theory (DFT) calculations

The vibrational dynamics of PTX, bio-NCP and bio-NCP + PTX were investigated by means of neutron spectroscopy using the FDS spectrometer at the Lujan Center of the Los Alamos National Laboratory (USA) at 10 K. By using this spectrometer it was possible to observe molecular motions between 50 and 500 cm^−1^. The samples were mounted in sealed aluminium containers and a vanadium run was used for correcting the data.

Structural optimization of the gas-phase paclitaxel molecule at 0 K and subsequent calculation of harmonic vibrational frequencies were carried out at the B3LYP/6-31 + G(d′) level of theory using Gaussian09^52^. The starting atomic positions were taken from the crystal structure of 2-carbamate taxol, described in ref. 53. Frequencies and vibrational amplitudes from the Gaussian calculation were then used to calculate intensities and vibrational spectra of the inelastic neutron scattering spectra using the program a_climax^54^. The FTIR spectrum for the PTX was also calculated and used for mode assignment in the range from 1400 to 1900 cm^−1^.

## Additional Information

**How to cite this article**: Martins, M. L. *et al*. Restricted mobility of specific functional groups reduces anti-cancer drug activity in healthy cells. *Sci. Rep.* **6**, 22478; doi: 10.1038/srep22478 (2016).

## Supplementary Material

Supplementary Information

Supplementary Movie S1

Supplementary Movie S2

Supplementary Movie S3

Supplementary Movie S4

Supplementary Movie S5

Supplementary Movie S6

Supplementary Movie S7

Supplementary Movie S8

Supplementary Movie S9
