# Supplementary material for: Restricted mobility of specific functional groups reduces anti-cancer drug activity in healthy cells
Source: Sci Rep. 2016 Mar 2;6:22478. doi: 10.1038/srep22478 (PMC4773877; doi:10.1038/srep22478)
Supplement: Supplementary Information [file srep22478-s1.pdf]

Supplementary Information

Restricted mobility of specific functional groups reduces anti-cancer drug activity in healthy cells

Murillo L. Martins[1,2], Rosanna Ignazzi[1], Juergen Eckert[3,4], Benjamin Watts[5], Ramon Kaneno[2], Willian F. Zambuzzi[2], Luke Daemen[4], Margarida J. Saeki[2] and Heloisa N. Bordallo[1,6*]

[1]Niels Bohr Institute, University of Copenhagen, DK-2100 Copenhagen, Denmark; [2]Instituto de Biociências - Universidade Estadual Paulista – CP 510, 18618-970 Botucatu – SP, Brazil; [3]Department of Chemistry, University of South Florida, 4202 E. Fowler Ave., Tampa, Florida 33620, United States; [4]Los Alamos National Laboratory, Los Alamos, New Mexico 87545, United States; [5]Swiss Light Source, Paul Scherrer Institute, CH-5232 Villigen, Switzerland; [6]European Spallation Source ESS AB, PO Box 176, SE-221 00 Lund, Sweden.

*Correspondence and requests for materials should be addressed to: H.N.B (bordallo@nbi.ku.dk)

Details about the sample preparation. Mn-Zn ferrites, $Mn_{1.05}Zn_{0.25}Fe_{1.70}O_4$, were prepared by the co-precipitation method as described in, [1] using $Mn(NO_2)_2$, $Zn(NO_2)_2$ and $FeCl_3$ as starting salts. The salts were diluted in water, dripped into boiling 0.1M NaOH solution, allowed to react for 120 min and the resulting precipitate was collected using a magnet and washed with distilled water. The resulting particles were then dispersed in water and a 40 mg/mL ferrite suspension was obtained. Then, the encapsulation of the magnetic nanoparticles together with the paclitaxel (PTX) was performed by a double emulsion method. To do so, 20 mg of chitosan were dissolved in 2 mL of 4% acetic solution. 1 ml of the Mn-Zn ferrite nanoparticles dispersion was added to the chitosan solution together with 0.25mL of the surfactant Tween 80. The resulting ferrite + chitosan dispersion was vigorously stirred for 30 min. Meanwhile, 25mg of PTX was dissolved in 0.5mL of dichloromethane. After 30 min, the PTX solution was added to the Mn-Zn ferrite + chitosan dispersion and stirred for 120 min. The resulting suspension of Mn-Zn ferrite + chitosan + PTX was added to an organic solution prepared with 50mL of paraffin and 3.17mL of oleic acid and stirred for 120 min. At this stage, 0.1 mL of glutaraldehyde (25%) was dripped into the solution to perform the cross-linking reaction of chitosan. The reactants were stirred for 120 min at room temperature and then kept at

70°C for 12h. The resulting material was carefully washed with ethanol, ether and acetone to ensure that the sample contained no paraffin and surfactants and re-suspended in 100mL of 0.5M $CaCl_2$ solution and allowed to stir for 120 min in order to perform the surface modification with apatite by the mimetization method [1]. Then, 20 mL of H_3PO_4 0.1% were added to the solution and the pH was adjusted to 7.4 with NaOH. An additional 60 min of stirring was conducted. Finally, the sample was separated with a magnet and dried at 60°C for 72h. In the main text, the sample containing the PTX encapsulated into the bio-NCP was called "bio-NCP + PTX". A sample without PTX was also prepared by following all the steps above and was referred to as "bio-NCP". All the reagents used in the procedures described here were purchased from Sigma-Aldrich.

Preparation of cell cultures *human monocytes, HCT116 (colon cancer), 3LL (lung cancer) and* Balb/c 3T3 fibroblasts. Human monocytes were isolated from 40 mL of peripheral blood of healthy donors collected with heparin-containing flasks. Blood samples were subsequently diluted in an equal volume of phosphate buffered salt solution (PBS) and the mononuclear cells obtained by centrifugation on a gradient of Ficoll-Isopaque solution (d=1,077 g/mL) for 30 min at centrifugation speed of 900x g. Afterwards, the cell suspension was washed three times with fresh RPMI 1640 culture medium, suspended in PBS containing 5% of fetal bovine serum (FBS) and centrifuged on a Percoll 51% gradient in order to separate most of monocyte (interface) from lymphocytes (pellet). The resulting monocyte-rich suspension, the HCT116 and the 3LL cells were then washed three times with RPMI 1640, supplemented with 10% FBS, 1% nonessential amino acids, 1% sodium pyruvate, 25 mM HEPES, 2mM L-glutamine, and 1% antibiotic/antimitotic solution (complete culture medium). Finally, the cells were set to 2 x 10^5 cells/mL and dispensed on rounded glass slides (∅13 mm), previously coated with poly-L-lysine.

Regarding the Balb/c 3T3 fibroblasts, the cells were cultured in a DMEM media containing $NaHCO_3$ (1.2 g/L), ampicillin (0.025 g/L) and streptomycin (0.1 g/L)

supplemented with 10% FBS (Cultilab) at 37 ºC in 5% CO_2 atmosphere. Afterwards the cells were dispensed in 96-well plates, each one containing $5x10^4$ cells, and kept in culture for 24h.

Infrared spectroscopy. Fourier transformed infrared spectra (FTIR) for the PTX and for the as prepared bio-NCP + PTX were obtained using an ATR Crystal (Bruker) with a 1.4 cm-1 resolution [2]. In addition, to verify the full release of the PTX 20mg of the bio-NCP+PTX was dispersed in aqueous media for 7 days. The sample was then dried under vacuum at room temperature for 72h. The FTIR spectrum of the resulting powder was collected using a Nicolet spectrometer, Nexus 670, with a 2 cm-1 resolution. The experimental and calculated spectra are shown in Figure SI1.

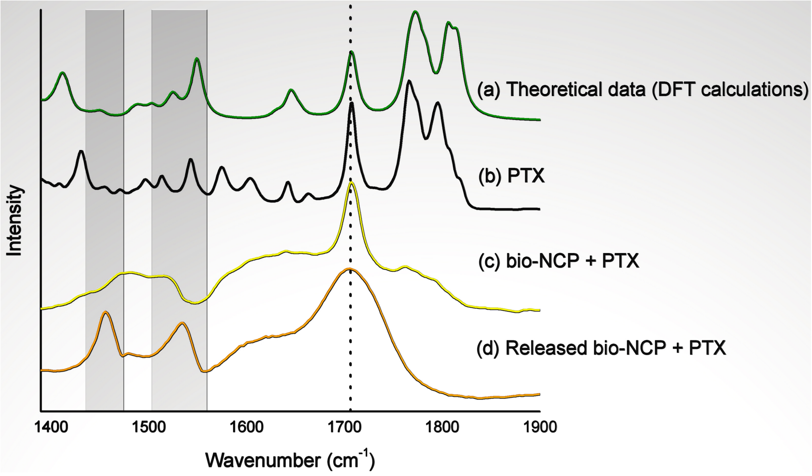

Figure SI1. (a) Theoretical and (b) experimental FTIR spectra for PTX, (c) spectra for the bio-NCP+PTX as prepared and (d) after being dispersed in water as described in the text. The spectra from pure PTX and bio-NCP+PTX were taken from [2] and were collected in a higher resolution instrument.

Mode assignment obtained using DFT calculations. The origin of the most relevant vibrational modes in PTX, Figure SI2, were determined from the frequencies and atomic displacements calculated by Density Functional Theory (DFT). These modes are listed in Table SI1.

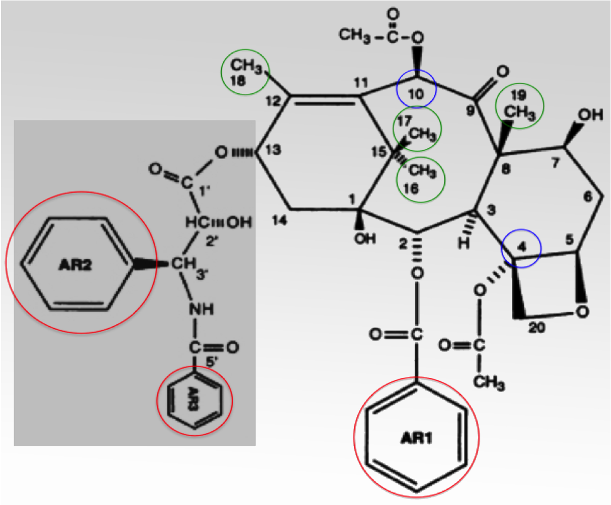

Figure SI2. Schematic structure of the PTX molecule adapted from [3]. Phenyl groups (AR1, A2 and AR3) are highlighted in red, methyl groups in green and carbons bonded to acetyl groups in blue. The dark grey shading highlights the flexible side chain bonded to C13.

Table SI1. Most relevant contributions from the PTX molecule to the INS vibrations obtained by DFT calculations. AR denotes the Aromatic Rings.

**Calculated frequency (cm^{-1})**	**Main contributions**
6.52	AR1, AR2, AR3
18.70	C13 side chain and AR1
29.24	AR1 and oxetane ring
45.39	AR1 and oxetane ring
52.89	AR2, AR3 and Acetyl 1
57.27	Acetyl 1
67.75	Acetyl 1 and AR1
73.79	Acetyl 1 and AR1
74.50	Acetyl 1, Acetyl 2, oxetane ring
85.48	Oxetane ring, methyl groups in C16, C17 and C19 and C6
95.60	Acetyl 2, methyl groups in C16, C17, C18 and C19, AR1 and C6

109.5	C14, AR3, Acetyl 2
130.7	C6, C7, oxetane ring and methyl in C17
140.4	Side chain in C13 and methyl groups in C16 and C17
184.5	Methyl groups in C17 and C18, AR3, Acetyl 2 and C14
205.3	C14 and methyl groups in C17 and C18
211.6	Methyl groups in C16, C17 and C19 and Acetyl 1
221.6	Methyl groups in C16, C17 and C19 and C14
225.8	Methyl in C19 and oxetane ring
253.5	Side chain in C13 and methyl in C18
271.0	C16, C17
273.4	Methyl groups in C16 and C19 and Acetyl 2
302.2	AR1, Acetyl 2 and methyl groups in C18 and C19
335.0	Methyl in C16, OH bonded to C7 and side chain in C13
387.5	OH bonded to C7 and side chain in C13
391.3	OH bonded to C7 and oxetane ring
411.2	OH bonded to C7, oxetane ring and methyl groups in C18 and C19

Movie S1. The movie shows the motions for the PTX molecule at 29cm^{-1} as obtained by DFT calculations.

Movie S2. The movie shows the motions for the PTX molecule at 57cm^{-1} as obtained by DFT calculations.

Movie S3. The movie shows the motions for the PTX molecule at 80cm^{-1} as obtained by DFT calculations.

Movie S4. The movie shows the motions for the PTX molecule at 109cm^{-1} as obtained by DFT calculations.

Movie S5. The movie shows the motions for the PTX molecule at 131cm^{-1} as obtained by DFT calculations.

Movie S6. The movie shows the motions for the PTX molecule at 205cm^{-1} as obtained by DFT calculations.

Movie S7. The movie shows the motions for the PTX molecule at 271cm^{-1} as obtained by DFT calculations.

Movie S8. The movie shows the motions for the PTX molecule at 1535cm^{-1} as obtained by DFT calculations.

Movie S9. The movie shows the motions for the PTX molecule at 1550cm^{-1} as obtained by DFT calculations.

[1] Martins, M. L. *et al*. Development and characterization of a new bio-nanocomposite (bio-NCP) for diagnosis and treatment of breast cancer. *J. Alloys Compd*. **584,** 514-519 (2014).
[2] Martins, M. L. *et al*. Encapsulation of paclitaxel into a bio-nanocomposite. A study combining inelastic neutron scattering to thermal analysis and infrared spectroscopy. *EPJ Web of Conferences*. **83,** 02011 (2015).
[3] Mastropaolo, D., Camerman, A., Luo, Y., Brayer, G. D. & Camerman, N. Crystal and molecular structure of paclitaxel (taxol). *Proc. Natl. Acad. Sci. USA* **92,** 6920-6924 (1995).
